# Preparation and Characterization of Furan–Matrix Composites Blended with Modified Hollow Glass Microsphere

**DOI:** 10.3390/polym12071480

**Published:** 2020-07-01

**Authors:** Yizhe Ma, Ying Du, Jin Zhao, Xubo Yuan, Xin Hou

**Affiliations:** School of Material Science and Engineering, Tianjin University, Tianjin 300350, China; 13132263022@163.com (Y.M.); 3013208005@tju.edu.cn (Y.D.); zhaojin@tju.edu.cn (J.Z.); xbyuan@tju.edu.cn (X.Y.)

**Keywords:** furan resin, hollow glass microsphere, thermal stability, thermal insulation material

## Abstract

In this study, a new class of thermal insulation composites was prepared by blending a modified hollow glass microsphere (HGM) with furan resin. The particle dispersion between the microparticles and resin matrix was improved using 3-methacryloxypropyltrimethoxy silane (KH-570). Furthermore, the structure and morphology of the modified HGM were characterised by Fourier transform infrared spectroscopy (FTIR), X-ray photoelectron spectroscopy (XPS), and scanning electron microscopy (SEM). In addition, the effects of the modified HGM on the thermal insulation, flame retardancy, and thermal properties of the composites were investigated. The thermal conductivity of the composites was lower than that of the native furan resin. The minimum thermal conductivity of the composites was 0.0274 W/m·K; the flame retardancy of the composites improved, and the limiting oxygen index become a maximum of 31.6%, reaching the refractory material level. Furthermore, the thermal analysis of the composites demonstrated enhanced thermal stability. This study demonstrates that the composite material exhibited good thermal insulation performance and flame retardancy and that it can be applied in the field of thermal insulation.

## 1. Introduction

Thermal insulation materials are developed to reduce heat transmission, which is based on bonding mechanical or chemical combination to decrease the heat transmission via various combined approaches (i.e., convection, radiation, and conduction) [1]. The thermal insulation materials are classified as organic insulation materials, inorganic insulation materials, and composite insulation materials according to the physical and chemical properties of the raw materials. Among them, the organic insulation materials are widely used because of their attractive characteristics [2]. However, some of the organic insulation materials, such as polyurethane and expanded polystyrene, exhibit significant drawbacks because of their inherent flammability [3,4]. In addition, the environmental concerns associated with the extensively used raw materials derived from fossil fuels have increased awareness with respect to the development of environmentally friendly materials [5]. Therefore, a flame-retardant and environmentally friendly thermal insulation material should be developed.

Furan resin is a typical bio-based thermosetting material [6,7] and is obtained from renewable resources [8,9]. Furfural, which is the raw material of furan resin, can be readily obtained from the agricultural residues containing pentose [10]. Furan resin is extensively used within the foundry industry and as wood adhesives [11,12], which exhibits great performance at flame retardancy, unlike most organic materials [13,14]. Furthermore, furan resin contains a non-toxic furfuryl group; therefore, the emission of contaminants can be avoided during its synthesis and application [15]. Accordingly, the usage of furan resin can reduce environmental pollution [16,17,18].

However, furan resin exhibits poor thermal insulation performance when compared with those exhibited by the traditional organic insulation materials. Generally, the thermal insulation efficiency of materials or composites can be enhanced by embedding the thermal insulation material in the resin matrix to resist heat transmission [19]. The hollow glass microsphere (HGM) is an inorganic particle filled with inert gas, and its hollow core results in excellent adiabaticity [20,21]. The HGM/resin composite is a type of closed-cell foam having a special structure that can be synthesised by dispersing HGM in the resin matrix. Liang et al. [22] prepared HGM/polypropylene composites; here, the thermal conductivity of the composite linearly declined as the HGM content increased. Furthermore, the thermal insulation effect of the composite became more obvious with the improved dispersion of the HGM in the resin matrix. Yung et al. [23] prepared HGM/epoxy composites and observed that the thermal conductivity of the composite declined as the HGM content increased. The remaining composite properties, including the glass transition temperature and thermal expansion coefficient, were also enhanced. Until now, few studies have investigated the HGM/furan resin composites. The HGM/furan resin composites can be used as a flame-retardant thermal insulation material because of the excellent thermal insulation properties and flame retardancy of the HGM [24].

**Figure 1:**
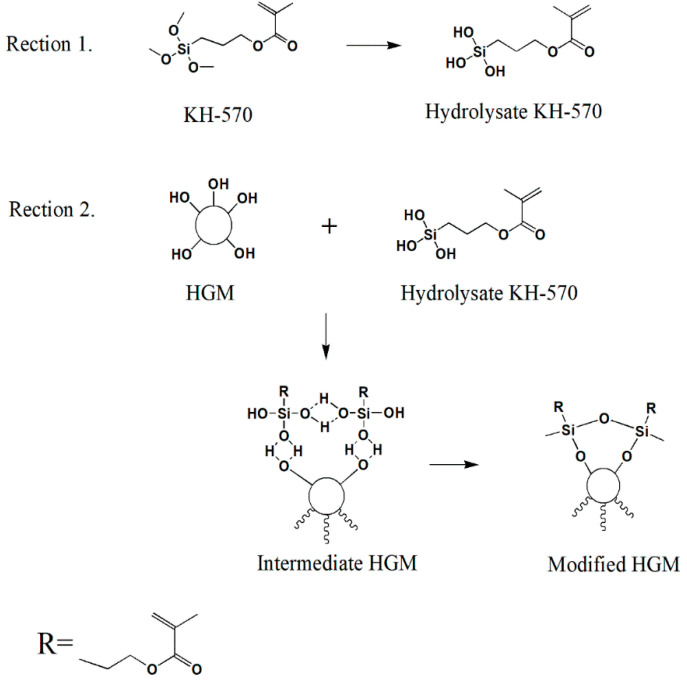
The hollow glass microsphere (HGM) surface modification mechanism using 3-methacryloxypropyltrimethoxy silane (KH-570).

In this study, the HGM/furan resin composite was developed. Since HGM is prone to agglomeration [25], 3-methacryloxypropyltrime-thoxy silane (KH-570) was added as the silane coupling agent to improve particle dispersion [26]. Figure 1 shows the reaction associated with HGM surface modification using KH-570. A scanning electron microscope (SEM) was used to characterise the morphology of the HGM and modified HGM. Fourier transform infrared spectroscopy (FTIR) and X-ray photoelectron spectroscopy (XPS) were used to analyse the grafting of KH-570 with HGM. Furthermore, the thermal performance, flame retardancy, and thermal insulation of the composites with respect to the modified HGM content were investigated.

## 2. Materials and Methods

### 2.1. Materials

The furan resin containing polyfurfuryl alcohol oligomers was supplied by Jinan Yisheng Resin Co. Ltd., Jinan, China. The resin had a water content of 5%, viscosity of 100 cps @ 25 °C and a density of 1.2 g/cm^−3^; KH-570 (boiling point = 255 °C) was used to modify HGM provided by The Kangjin New Material Technology Co. Ltd., Dongguan, China. The curing agent, i.e., benzenesulfonic acid (trade name: NL curing agent; total acidity = 30% ± 2% [by H_2_SO_4_]; free acidity = 6% ± 1% [by H_2_SO_4_]), was supplied by The Jining Hongming Chemical Reagent Co. Ltd., Jining, China. The HGM was obtained from The Huzhou Prospect Chemical Pharmaceutical Co. Ltd., Huzhou, China.

### 2.2. Sample Preparation

Initially, the HGM was placed in a vacuum drying box and dried at 80 °C for 24 h. Subsequently, the HGM was added into a mixture of ethanol and water (3:1) with KH-570 (1 wt % of HGM) and heated at 75 °C, followed by washing using distilled water and drying at 80 °C in vacuum conditions.

The furan resin and modified HGM were blended in a specific mass ratio (the content of modified HGM was 5, 10, 15 and 20 wt % of the furan resin). The mixture was subjected to 6 h of stirring at 75 °C. Subsequently, curing agent was added to the mixture. Then, the solution was poured into the mould, curing at 25 °C for a period of 24 h, and then another period of 2 h at 80 °C. The neat furan resin samples and HGM/furan resin composites were simultaneously prepared for the control group. The investigated materials are presented in Table 1.

### 2.3. Characterization

The morphology of the materials was investigated using SU-1510 SEM (Hitachi High-Technologies, Tokyo, Japan), and the electron beam voltage was maintained constant at 5 and 15 kV. Each sample was sputter-coated with gold prior to analysis. The Nicolet 6700 FTIR spectrometer (Thermo Nicolet, Madison, WI, USA) was used to obtain the FTIR spectra of the HGM and the modified HGM. The surface elemental content of the HGM and modified HGM was determined using the ESCALAB 250Xi XPS analyser (Thermo Fisher Scientific Inc., Waltham, MA, USA). Further, the composites were subjected to thermogravimetric analysis (TGA) using a Q-50 instrument (TA Instrument, New Castle, DE, USA) in a nitrogen atmosphere. The samples were subjected to heating at a rate of 10 °C/min at temperatures of 30–800 °C. Each sample was evaluated thrice to obtain the statistical significance. Dynamical mechanical analysis (DMA) was performed using a dynamic mechanical analyser (DMA Q800) (TA Instrument, New Castle, DE, USA) at frequency and heating rates of 1 Hz and 10 °C/min, respectively. The TC3000 thermal conductivity instrument (Xi’an Xiatech Electronic Technology Co. Ltd., Xi’an, China) was used for determining the thermal conductivity of the composite. Three independent tests were performed to obtain the reproduced data. The HC-2C oxygen index machine (Jiangning Analytical Instrument Co., Jiangning, China) (with sheet dimensions of 100 × 6.5 × 3 mm^3^) was used in the limiting oxygen index (LOI) test; the final LOI value was the average of the value obtained via three repeated tests. The CFZ-2-type device with dimensions of 130 × 13 × 1.5 mm^3^ was used for the UL-94 vertical burning test, with classifications of V-0, V-1, and V-2. The UL-94 rating was obtained by testing the three samples.

## 3. Results and Discussion

### 3.1. Characterizations of HGM and Modified HGM

**Figure 2:**
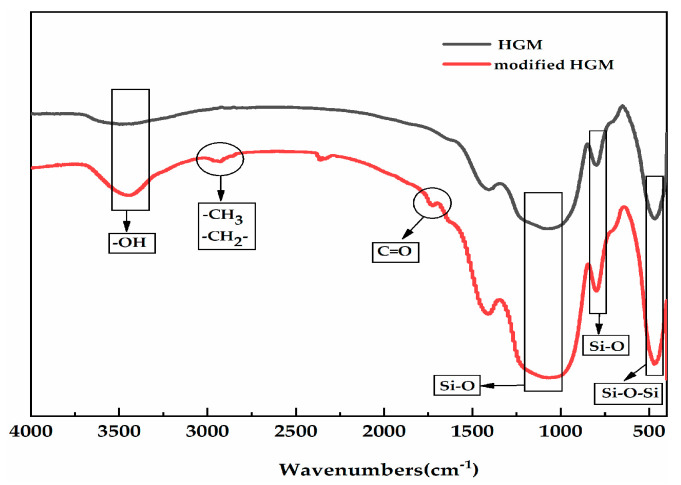
The Fourier transform infrared spectroscopy (FTIR) spectra of hollow glass microsphere (HGM) and modified HGM.

FTIR, XPS, and SEM analyses were performed to investigate the combination of HGM with KH-570. Figure 2 shows the FTIR spectra of HGM and modified HGM. The absorption peak at 3420 cm^−1^ could be attributed to the physical adsorption of water in materials and hydroxide on the HGM surface. The characteristic adsorption peaks of silicon dioxide (SiO_2_; primary component of HGM) at 473, 800, and 1071 cm^−1^ can be attributed to the bending vibration of Si–O–Si (first vibration), the symmetric stretching vibration, and the asymmetric stretching vibration of Si–O (the latter two vibrations), respectively. Furthermore, the peaks that appeared at 2850 and 2930 cm^−1^ can be attributed to the corresponding overlap of the methyl and methylene anti-symmetric vibration absorption bands on the silane coupling agent [27,28]. The peak at 1742 cm^−1^ was the characteristic C=O group peak, which could be observed within the spectrum of the modified HGM, confirming the successful combination of KH-570 and HGM.

**Figure 3:**
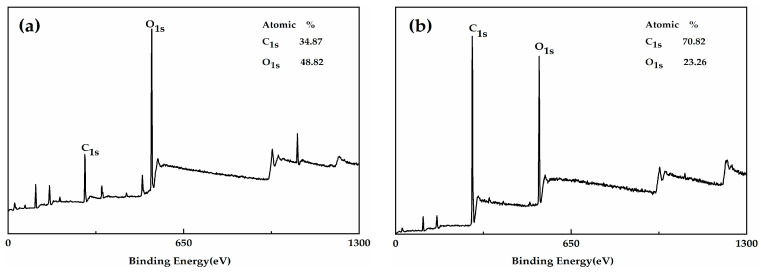
The X-ray photoelectron spectroscopy (XPS) spectra of (**a**) HGM and (**b**) modified HGM.

**Figure 4:**
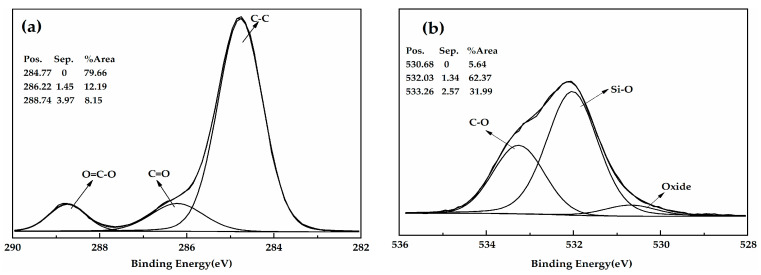
(**a**) The C_1s_ XPS spectra and (**b**) O_1s_ XPS spectra of the modified HGM.

Figure 3 shows the XPS spectrum of the HGM and modified HGM. The carbon content of HGM was only 34.87%, whereas that of the modified HGM was 70.82%. The presence of alkyl chains in KH-570 was the primary reason for the observed increase in carbon content. Figure 4 shows the C_1s_ and O_1s_ XPS spectra of the modified HGM. Three types of carbon can be observed in different chemical environments based on the C_1s_ XPS spectrum for the modified HGM. The binding energy at 284.77 eV represents the carbon in C–C, accounting for 79.66% of the total carbon content. The binding energies at 286.22 and 286.74 eV denote the carbon within the C=O–O and C–O bonds, respectively. Based on the O_1s_ XPS spectrum of the modified HGM, the binding energy of 532.03 eV represents the oxygen in Si–O with a content of 62.37%, which originated from SiO_2_ and the dehydration reaction between the hydroxyl groups on the surface of the HGM and the silanol groups formed by the hydrolysis of the coupling agent.

**Figure 5:**
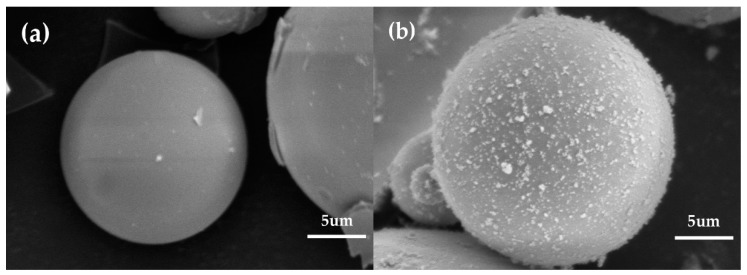
The SEM micrographs of (**a**) HGM and (**b**) modified HGM (magnification scale: ×4000).

Figure 5 shows the SEM images of the HGM and modified HGM. The HGM showed a smooth clean surface before modification, which would not promote the adhesion at the interface between the polymer matrix and inorganic particles [29]. However, the surface of the modified HGM was rough, which may be attributed to the introduction of alkyl chains associated with KH-570. The presence of alkyl groups can effectively reduce the overall polarity of the microspheres and improve their hydrophobicity, which is conducive for reducing the agglomeration between the microsphere.

### 3.2. Thermal Properties of the Composites

**Figure 6:**
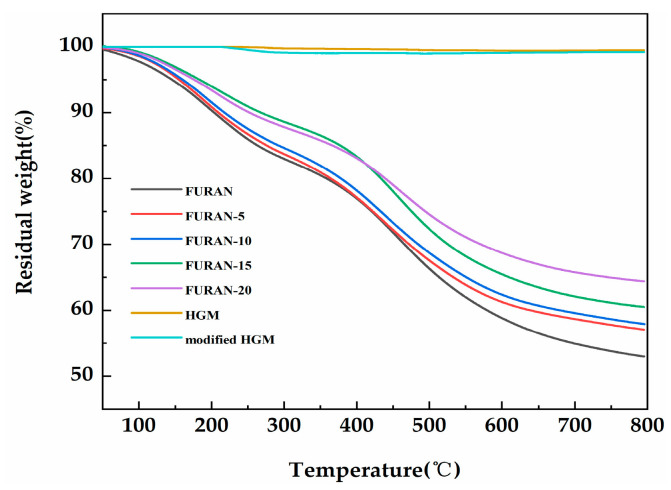
The thermogravimetric analysis (TGA) curves of the HGM, modified HGM, and modified HGM/furan resin composites.

**Figure 7:**
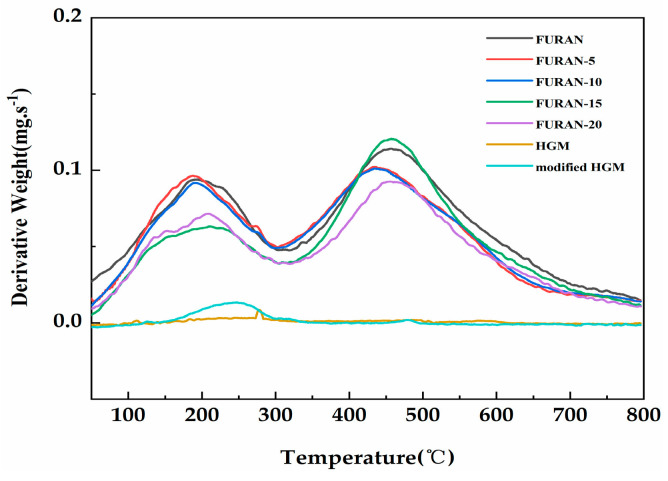
The derivative thermogravimetric (DTG) curves of the HGM, modified HGM, and modified HGM/furan resin composites.

The thermogravimetry in a nitrogen (as opposed to air) atmosphere is considered to be an appropriate flame feeding model during the polymer combustion process because most of the oxygen is used up for the flame, and only a small portion can reach the polymer surface [30]. Figure 6 shows the TGA curves of the HGM, modified HGM, and composites in nitrogen. Table 2 presents the temperature results of composites involving weight losses of 5 wt % and 10 wt % (T_5%_ and T_10%_, respectively) and char production at a temperature of 800 °C. The decomposition temperature of each composite increased compared with that in the pure furan resin. The initial thermal stability of the composites improved with the increasing T _5%_ and T _10%_. The modified HGM did not significantly affect the weight loss curve of the furan resin; however, the mass residual rate of the composite material increased from 53% to 64.3% when the modified HGM content increased from 0% to 20%. Furthermore, compared with the HGM, the modified HGM experienced only a tiny mass loss at 255 °C because of the decomposition of alkyl chains on the HGM’s surface. The thermal degradation curves of the HGM and modified HGM maintained a stable trend with the increasing temperature until 800 °C, exhibiting an excellent thermal stability similar to that exhibited by HGM. Figure 7 shows the corresponding derivative thermogravimetric (DTG) curves of the HGM, modified HGM, and composites. Two degradation links were observed from the DTG curves in case of composites. Degradation was mainly observed because of impurity (water) volatilisation, residual monomers, and oligomers from 0 to 300 °C. The second degradation link could be observed from 400 to 600 °C, where the cross-linked structural fragmentation resulted in volatile and combustible chemical moieties. For temperatures higher than 600 °C, the structure decomposed into carbonaceous residue or coke [31]. HGM remained stable throughout the process without any decomposition, whereas the modified HGM underwent a tiny mass loss at 255 °C because of the decomposition of alkyl chains on the HGM’s surface. Generally, the thermal stability of a material was improved by the incorporation of modified HGM.

**Figure 8:**
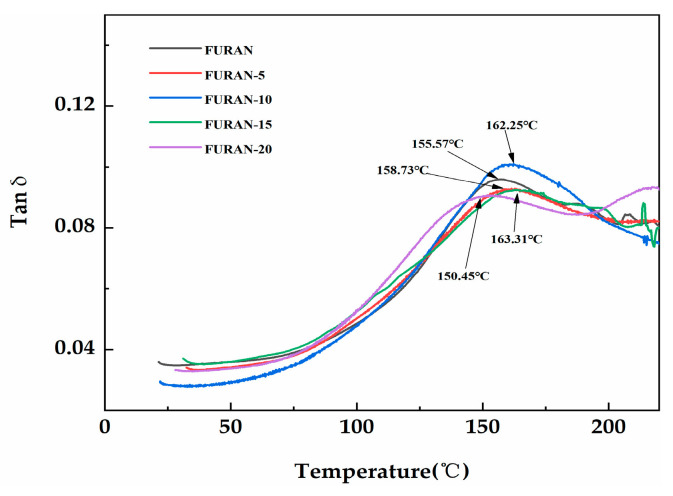
The dissipation factor (tan δ) of the modified HGM/furan resin composites.

DMA is an extremely sensitive technique used to determine the viscoelastic behaviour of polymers and can help to understand the associations among the cross-linked material morphology, structure, and properties. In addition, the loss factor tan δ in DMA is defined as the loss modulus/storage modulus ratio, indicating high sensitivity to the solid structural transformation of material. T_g_ was calculated based on the tan δ loss factor peaks [11]. The temperature dependence of tan δ with respect to various composites is presented in Figure 8. As shown in Figure 8, the T_g_ of pure furan resin was only 155.57 °C. As the modified HGM content increased, the T_g_ of the composites became 163.31 °C when the modified HGM content became 15 wt %. The addition of micrometre-sized inorganic particles increases the T_g_ of the composite, which is induced by the association between the particles and polymer phases, thereby restricting the polymer chain mobility [32]. However, the curing reaction of the resin may be affected, and the cross-linking density of the system was reduced when the modified HGM was considerably high, decreasing the T_g_ [33].

**Figure 9:**
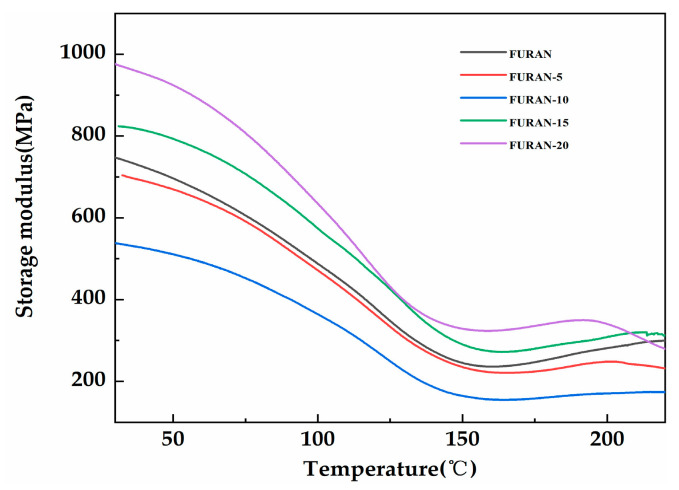
The storage modulus of the modified HGM/furan resin composites.

Figure 9 presents the variation in composite storage modulus based on the temperature changes. As shown in Figure 9, the composite storage modulus initially showed a decreasing trend, followed by gradual stabilisation with the increasing temperature. As the temperature increased towards the transition zone, the synergistic effect of the intermolecular segments of the matrix caused the storage modulus to drastically decrease. Once the temperature became higher than 150 °C, the system reached the rubber zone and the storage modulus of the composites tended to reach a stable state at low values. When the content of modified HGM was low, the composites showed a reduced storage modulus because of the interruption effect of modified HGM in the network, reducing the network density of the composites [34]. However, as the content of the modified HGM became greater than 10 wt %, the large amount of inorganic rigid particles resulted in considerable material rigidity in the low-temperature glass region, increasing the corresponding storage modulus [35].

### 3.3. Thermal Insulation Characteristics of the Composites

**Figure 10:**
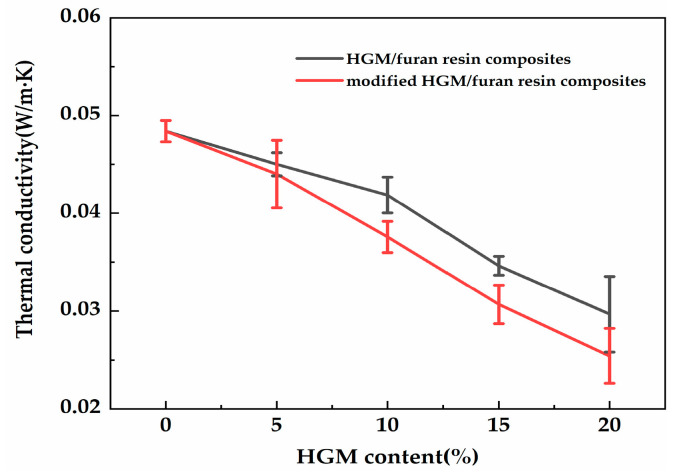
The thermal conductivity of the HGM/furan resin composites and modified HGM/furan resin composites.

**Figure 11:**
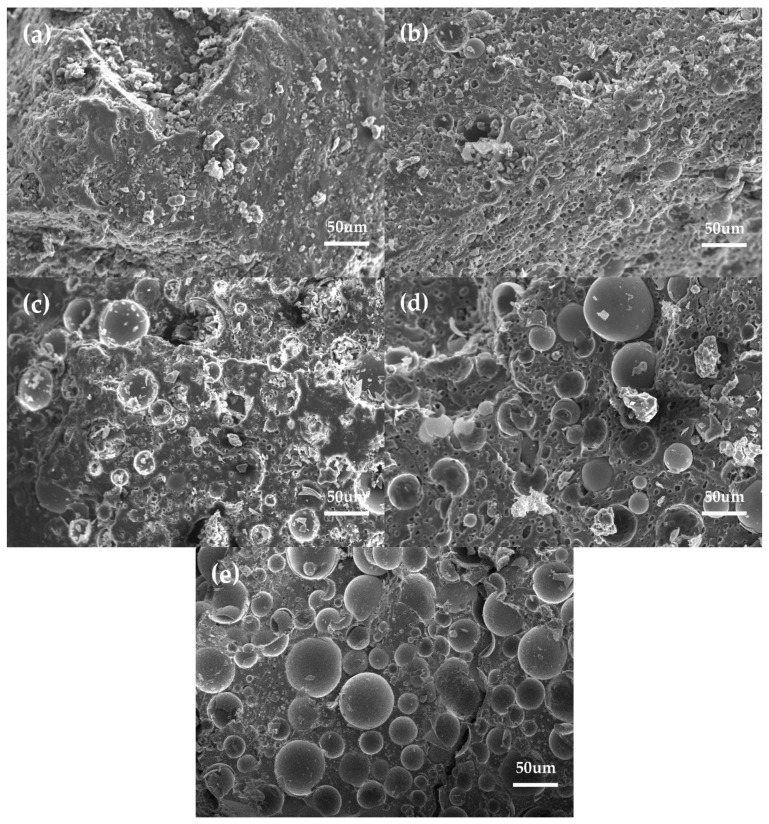
The SEM micrographs of the modified HGM/furan resin composites. (**a**) FURAN, (**b**) FURAN-5, (**c**) FURAN-10, (**d**) FURAN-15, and (**e**) FURAN-20 (magnification scales: ×300).

Based on the thermal conductivity of the composite, high-quality thermal insulation materials could be obtained. Figure 10 shows the thermal conductivity of the composite with different HGM and modified HGM content. As shown in Figure 10, the thermal conductivity of the composite decreased as the modified HGM content increased. Neat furan resin had a thermal conductivity of 0.048 W/m·K, whereas the thermal conductivity of the composite declined to 0.025 W/m·K when 20 wt % modified HGM was added. The inert gas in the interior resulted in the reduced thermal conductivity of the modified HGM [21]. Consequently, the thermal conductivity of the composite declined as the modified HGM content increased. HGM/furan resin composites were also prepared to study the effect of KH-570 on the thermal conductivity of the composites. The thermal conductivity of the HGM/furan resin composites decreased with the increasing HGM content. However, the decreasing trend was not as significant as that of the modified HGM/furan resin composites. When 20 wt % HGM was added, the thermal conductivity of the composite became higher than that of the modified HGM/furan resin composite. Therefore, the thermal insulation effect of the composite will increase with the increasing modified HGM content. The uniform distribution of modified HGM within the matrix contributed to improve the composite’s thermal insulation performance, particularly when there was a high degree of modified HGM [22]. Figure 11 shows the SEM images of the modified HGM/furan resin composites. No obvious aggregation of the modified HGM could be observed in the micrographs of the modified HGM/furan resin composites, indicating the considerably uniform dispersion of modified HGM within the matrix. Furthermore, the majority of the modified HGM could be observed close to the resin and would not fall on the resin surface.

### 3.4. Flame Retardancy of the Composites

**Figure 12:**
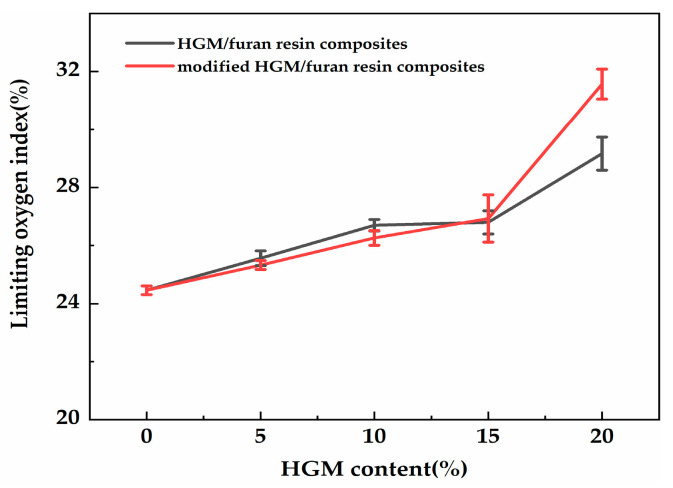
The limiting oxygen index (LOI) values of the HGM/furan resin composites and the modified HGM/furan resin composites.

**Figure 13:**
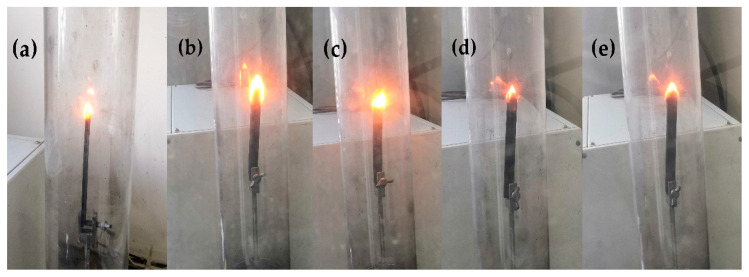
The combustion morphologies of the modified HGM/furan resin composites in the test: (**a**) FURAN, (**b**) FURAN-5, (**c**) FURAN-10, (**d**) FURAN-15, (**e**) FURAN-20.

The UL-94 vertical burning test and LOI were considered to be the standard analysis methods for verifying the fire safety of materials. The LOI test refers to the minimal proportion of oxygen within the oxygen–nitrogen mixture that can sustain the combustion of a specimen after ignition [24]. The flame retardancy of the composites was preliminarily investigated via the UL-94 test and by establishing LOI values (Figure 12). The LOI value changed with the increasing modified HGM content. Regardless, when 5 and 10 wt % modified HGM were added, the composite UL-94 rating was identical to that of furan with a No rating, indicating that the composite did not meet the standard of the UL-94 test. The composite’s LOI value increased to 27.0% only when 15 wt % modified HGM was added, and the UL-94 test resulted in a V-1 rating. Additionally, the composite’s LOI value was the largest (31.6%) after the addition of 20 wt % modified HGM, and the UL-94 test resulted in a V-0 rating. The relevant properties of the HGM/furan resin composites were also analysed. As shown in Figure 12, the LOI value of the composite increased with the increasing in HGM content. When the content of inorganic particles was less, the LOI values of the HGM/furan resin composites and the modified HGM/furan resin composites were similar; however, when the content of inorganic particles was increased, the LOI value of the modified HGM/furan resin composite became slightly higher than that of the HGM/furan resin composite. The good dispersibility of inorganic particles and the compatibility of the modified HGM and furan resin will improve the flame retardancy of the composite [36]. Figure 13 presents the morphology of the initial combustion phase of the modified HGM/ furan resin composites. When the content of modified HGM was increased, the degree of combustion flame of the composites gradually decreased; thus, the addition of modified HGM would effectively improve the flame retardancy of the composite. Since SiO_2_ is the main component of modified HGM, the Si–O bonds can form an oxygen barrier to serve as an insulation layer on the material surface. Further, the char residue structure of the composite can be improved; consequently, a dense char residue was formed on the polymer surface to serve as a protective layer, improving the overall flame retardancy [24,37].

## 4. Conclusions

In this study, a new class of thermal insulation composite was prepared by blending modified HGM with the furan resin. Further, the effect of the modified HGM content on the performance of a furan–matrix composite was extensively examined. The addition of modified HGM effectively improved the flame retardancy and thermal insulation of the composite materials. Compared with the neat furan resin, the UL-94 test of the composite material achieved the V-0 rating, with the lowest thermal conductivity being 0.0274 W/m·K. Based on the thermal decomposition behaviour of the composite, the addition of modified HGM was observed to improve its thermal stability. According to these observations, the performance of the composites was generally enhanced. Thus, this type of material has application prospects in the field of thermal insulation and exhibits excellent thermal insulation and flame-retardant properties.

## Figures and Tables

**Table 1:** Modified HGM/furan resin composites and HGM/furan resin composites.

Sample	Furan Resin/g	Modified HGM/g	HGM/g
FURAN	10	0	0
FURAN-5	10	0.5	0
FURAN-10	10	1.0	0
FURAN-15	10	1.5	0
FURAN-20	10	2.0	0
FURAN-N5	10	0	0.5
FURAN-N10	10	0	1
FURAN-N15	10	0	1.5
FURAN-N20	10	0	2

**Table 2:** TGA data (N_2_) of the modified HGM/furan resin composites.

Material	T _5%_/°C	T _10%_/°C	Mass Residue/%
FURAN	145.4	203.5	53.0
FURAN-5	155.3	209.3	57.0
FURAN-10	160.1	217.6	57.9
FURAN-15	183.4	267.8	60.5
FURAN-20	176.0	252.1	64.4
